# Implications of Behavioral Architecture for the Evolution of Self-Organized Division of Labor

**DOI:** 10.1371/journal.pcbi.1002430

**Published:** 2012-03-22

**Authors:** A. Duarte, E. Scholtens, F. J. Weissing

**Affiliations:** Theoretical Biology, Centre for Ecological and Evolutionary Studies, University of Groningen, Groningen, The Netherlands; Indiana University, United States of America

## Abstract

Division of labor has been studied separately from a proximate self-organization and an ultimate evolutionary perspective. We aim to bring together these two perspectives. So far this has been done by choosing a behavioral mechanism *a priori* and considering the evolution of the properties of this mechanism. Here we use artificial neural networks to allow for a more open architecture. We study whether emergent division of labor can evolve in two different network architectures; a simple feedforward network, and a more complex network that includes the possibility of self-feedback from previous experiences. We focus on two aspects of division of labor; worker specialization and the ratio of work performed for each task. Colony fitness is maximized by both reducing idleness and achieving a predefined optimal work ratio. Our results indicate that architectural constraints play an important role for the outcome of evolution. With the simplest network, only genetically determined specialization is possible. This imposes several limitations on worker specialization. Moreover, in order to minimize idleness, networks evolve a biased work ratio, even when an unbiased work ratio would be optimal. By adding self-feedback to the network we increase the network's flexibility and worker specialization evolves under a wider parameter range. Optimal work ratios are more easily achieved with the self-feedback network, but still provide a challenge when combined with worker specialization.

## Author Summary

In insect colonies, different individuals specialize in different tasks related to colony maintenance and growth. Unveiling why this division of labor evolved and how individuals decide which task to take on is crucial for our understanding of complex group behavior. Here we model the evolution of general behavioral rules for processing environmental signals of task need in social insect colonies, using artificial neural networks. We examine the patterns of individual specialization that arise in the course of evolution. Division of labor is likely to evolve if switching between tasks decreases worker productivity, but the pattern of division of labor across colonies is highly dependent on the architecture of the networks considered. In networks that allow for a feedback of previous experience on future task choice, division of labor can evolve across the whole population of colonies. In networks where this feedback is not allowed, the presence of division of labor is constrained by the specific genetic composition of colonies. Network architecture also affects how fine-tuned the worker allocation to different tasks can be when the tasks have different requirements.

## Introduction

Division of labor is ubiquitous in nature. The major evolutionary transitions, such as the separation of germ and soma and the transition from prokaryotes to eukaryotes, were accompanied by an increase in division of labor [1]. The transition from solitary to eusocial in insects encompasses the evolution of a reproductive caste and a sterile worker caste. Furthermore, division of labor among sterile workers also evolved, in which different groups of workers specialize in different functions, such as foraging and brood care [2]. Colony growth and survival is strongly dependent on the coordinated interaction of a large number of workers. This non-reproductive division of labor is therefore often considered a major determinant of the ecological success of eusocial insects and will be the focus of the work presented here.

Empirical evidence suggests that eusociality has evolved in associations of close kin [3], [4]. Variation in behavioral tendencies can be found in forced associations of non-social individuals, leading to incipient forms of division of labor [5], [6]. Undoubtedly, a source of variation is key to generating consistent inter-individual differences and task specialization [7]. The questions that arise are how and why such variation arises among close kin. Here we explore some of the mechanisms and conditions through which task specialization can evolve in groups of related individuals.

Recent work on division of labor in insect societies has focused on the self-organization properties of colony behavior. According to a variety of models [8]–[12] colony properties emerge from the behavior of individual workers whose reactions to the environment is governed by simple rules. The behavioral rules leading to emergent specialization are probably shaped by natural selection [10], [13], yet only few studies have focused on the evolution of these rules [14], [15]. Previous work focusing on the benefits of task specialization in other systems (e.g. enzyme-substrate specialization, coordination in co-viruses) generally disregard the mechanisms underlying it, viewing instead specialists and generalists as fixed behavioral strategies [16], [17]. It is thus important to develop models that integrate the evolutionary and self-organization perspective, in order to create a better understanding of division of labor and its evolution [7].

In previous work, we took the response threshold model [1] as a starting point for an evolutionary model for division of labor (A. Duarte, I. Pen, L. Keller and F.J. Weissing, submitted). In the response threshold model, individuals compare an environmental stimulus for a task with their response thresholds; they perform the task if the stimulus is above their threshold, otherwise they remain idle. Using this predefined behavioral architecture, we allowed the evolution of threshold values and showed that division of labor can evolve from a homogeneous population via evolutionary branching, but only if there are clear fitness benefits of individual specialization. Our work also revealed that the response threshold model has the drawback that it imposes severe constraints on the distribution of workers over tasks.

Here we look at a more flexible behavioral architecture that is represented by a simple artificial neural network (ANN). ANNs simulate the processing of stimuli by individuals, from stimulus perception by receptor nodes to effector nodes determining the behavioral output [18], [19]. ANNs have been used in evolutionary robotics to understand the evolution of communication and cooperation [20]–[22]. In a recent paper, Lichocki et al. showed that ANN's, in comparison to response threshold mechanisms, allow for more efficient worker allocation through task switching [23]. Here we examine the effect of the architecture of ANN's in worker specialization and worker allocation, in a context where task switching is detrimental.

**Figure 1:**
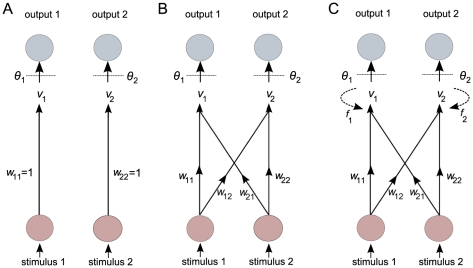
Architecture of the three types of networks. Stimulus values are perceived by input neurons. The stimuli are then processed by the network, resulting in an activation energy 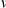 for each output neuron. An output neuron is excited whenever the activation energy is larger than the neuron's threshold 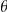. (A) Feedforward neural network, equivalent to the architecture encapsulated in the response threshold model, where only weights *w*_11_ and *w*_22_ exist. Hence, the activation energy is equal to the perceived stimulus. (B) Feedforward neural network, fully connected. (C) Recurrent neural network, where self-feedback occurs between activation energies of previous time steps and current activation energies.

In the response threshold model, the response to task-associated stimuli is determined by task-associated thresholds. The stimuli, which reflect the colony's need for work on the various tasks, change dynamically due to two factors: there is an inherent tendency for the stimuli to increase, and they are decreased whenever the corresponding task is performed. We keep most assumptions of the threshold model but allow the task-associated stimuli to be processed by an ANN. In principle both the architecture of the network and the way information is processed could evolve [24], [25], however, we for simplicity, we focus on predefined architectures (with a fixed number of receptor and effector nodes) and allow only for the evolution of connections between the nodes. The stimuli are processed by an ANN consisting of two receptor nodes and two effector nodes (Figure 1). In a second part of our study, we keep the same network structure but allow for the evolution of a feedback from the effector nodes to the processing of the stimuli (Figure 1C). In other words, an effect of previous experience on current decisions can evolve. An effect of previous experience on task preference, leading to division of labor, has been observed in natural colonies [26], thus it would be interesting to observe under which circumstances it could evolve.

We investigate if these slightly more sophisticated mechanisms for processing input allow for the evolution of adaptive division of labor. More precisely, we study whether task specialization among workers can evolve and moreover, whether an appropriate distribution of workers over tasks can be achieved. Throughout, the main question is whether, and to what extent, the evolution of self-organized division of labor is determined by the underlying architecture of behavior.

## Model

The general aspects of the model follow A. Duarte, I. Pen, L. Keller and F.J. Weissing (subm.). We consider a population of 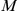 colonies, each founded by a single-mated individual that produces 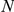 workers (typically 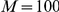, 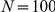). Each colony goes through a work phase consisting of 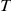 time steps (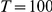), where all individuals perceive stimuli associated with two tasks and decide whether to perform one of the tasks or remain idle. The amount of work performed and the distribution of workers over tasks determines the fitness of a colony, which corresponds to the number of reproductives produced. Selection occurs because the colonies of a given generation are founded by pairs of reproductives produced in the previous generation. Hence colonies where the workers perform their tasks in the most efficient and coordinated way spread the genes of their foundresses most effectively.

In line with [8], we assume that there are two tasks and two task-associated stimuli. Stimuli increase each time step by a fixed amount 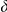 and decrease by an amount 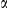 whenever a worker performs the task, following [8] (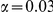 and 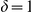 in our simulations). In the response threshold model, the association between stimuli and task was also expressed in the fact that individuals were more likely to perform a task for which the stimulus was high. However, in the present model, this is not necessarily the case. An association between task and stimulus is present because the performance of a given task decreases a given stimulus. Workers are assessed in random order and, once an individual works, the corresponding stimulus value is immediately decreased, such that the next worker to be assessed experiences a different stimulus value.

### Artificial neural networks

The first network studied is a simple feedforward network [19] that consists of two stimulus input nodes and two behavioral output nodes, all four nodes being connected (Figure 1B). Each input node perceives a task-associated stimulus with a certain error 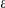 (drawn from a normal distribution with mean 0 and standard deviation 1). The two signals are then processed and transmitted to the output neurons, via connections with weights 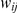 that are evolvable properties of the network. Output nodes receive a weighted sum of the stimuli, generally designated activation energy. The activation energy 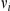 of an output node *i* is thus:
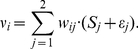
(1)Each output neuron is characterized by a threshold 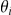, which is another evolvable property. If the activation energy of an output neuron exceeds the threshold, the neuron is activated, meaning that an individual is willing to perform the respective task. If both output neurons are activated, one task is chosen at random. Note that the response threshold model implemented in previous work is in fact a special case of the feedforward neural network, where 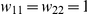 and 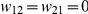 (Figure 1A). The main difference between our feedforward ANN model and the response threshold model is thus the evolution of the connection weights that determine how incoming information is processed and interpreted. The initial values of connection weights in our simulations are: 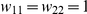 and 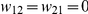. Changes in the connection weights and thresholds take place when new individuals are produced, via mutation (see below). During the lifetime of an individual, the parameters of its network are fixed. Thus we do not consider the changing of connection weights with learning, for example.

The second network architecture studied is a recurrent network [19]. It includes all previous nodes and connections, and in addition it has two self-feedback loops (Figure 1C). The activation energy in a given time step will affect the activation energy in the next time step: 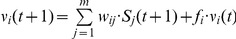. The connection weight 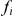 given to the previous activation energy (from here on called the self-feedback connection) is also an evolvable property that changes through mutation and natural selection during production of new individuals. During the lifetime of individuals, however, there is no change occurring in the parameters of the networks. Self-feedback connection weights were initialized at zero, which is equivalent to the feedforward network, without any influence of past experience in current decisions.

### Fitness

After the work phase, the fitness of each colony is computed based on how much work the workers performed for each task. Fitness is assumed to be proportional to the weighted geometric mean of work done for both tasks:
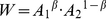
(2)where 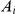 is the total number of acts performed for task 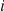 (A. Duarte, I. Pen, L. Keller, F.J. Weissing, subm.). We take the geometric rather than the arithmetic mean in order to ensure that fitness can only be achieved if both tasks are being performed. The weighing factor 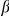 allows us to consider the (realistic) situation that not all tasks need to be performed equally often. For the fitness function (2), fitness is maximized if idleness is eliminated (*i.e.*, if 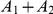 is maximal) and if the workers distribute over tasks according to the ratio 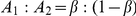. In other words, to maximize fitness the proportion 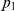 of work allocated to task 1 by the colony should be equal to 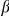:
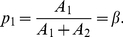
(3)


Each generation, 2*M* reproductive offspring are produced in total in the population. Colonies contribute to the population's pool of sexual individuals in proportion to their fitness. Population size is thus fixed. The reproductive individuals then form *M* pairs randomly. From each pair one individual will found a new colony with 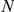 workers, while the old colonies are eliminated.

### Genetic details

We allowed for the evolution of all connection weights and thresholds of output nodes, giving us in a total 6 (resp. 8) evolving traits. These traits are encoded by 6 (resp. 8) gene loci. The alleles at these loci correspond to real numbers, with threshold alleles being larger or equal to zero, while connection weight alleles may also attain negative values. To keep the genetic assumptions as simple as possible, we assume that all individuals are haploid and that the network of each individual is fully determined by its genotype.

Genotypes of workers and sexuals are similarly inherited: Both types of individuals are offspring of the mated colony foundress, and possess alleles for thresholds and connection weights. Our model allows genetic linkage of the threshold loci or linkage of the connection weight loci, but both types of loci are considered to be sufficiently far apart in the genome to make them segregate independently. The degree of linkage is determined by a parameter 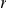 (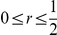) that corresponds to a recombination rate. With probability 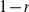, the threshold alleles (resp. the connection weight alleles) are inherited as a block from one of the two parents; with probability 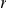, the parent whose allele is transmitted is chosen independently of what happens at the other loci.

Mutation occurs with probability 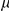 at each locus; when a mutation occurs, the genetic value at that locus is changed by adding a real number to it that is drawn from a normal distribution with mean 0 and standard deviation 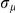. In our simulations, we typically used 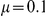 and 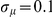.

### Measuring worker specialization

We evaluate colony-level characteristics such as the proportion of work devoted to each task and the level of individual specialization. For each individual we calculate at the end of a simulation the fraction 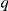 of time steps that it stayed in the same task from that time step to the next. We average 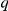 over all workers and normalize this measure by dividing 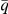 by the probability that individuals stay in the same task merely due to chance. The latter is given by 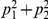, where 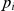 is the proportion of work devoted to task 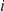. By subtracting 1 from the value thus obtained, we obtain a measure of worker specialization that ranges between −1 and 1 (A. Duarte, I. Pen, L. Keller and F. J. Weissing, subm.):
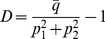
(4)When 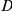 is close to 1, there is a high degree of division of labor, and individuals stay in the same task much more often than expected by chance. If 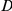 is close to zero, workers switch between tasks at random. If 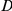 is lower than zero, individuals switch task more often than expected by chance.

### Switching costs

Worker specialization can be adaptive if there is a cost to switching tasks (such as a time cost if tasks are confined to different locations, or a cognitive cost), or if specialized workers perform their task with higher efficiency [27]. Here we implemented a time cost scenario, by imposing 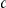 time steps of inactivity whenever an individual chooses to switch from one task to the other.

## Results

Simulations of the neural network model, with different network architectures were ran for 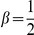, 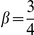 and switching costs 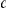 ranging from 0 to 5 time steps. We also tested the influence of recombination between the loci coding the neural network in the evolution of specialization. There were 10 replicates per parameter combination. The evolutionary patterns of the components of the neural networks were examined (thresholds of output neuron and connection weights) at the population level. Overall, connection weights were far more important than the thresholds in determining the behavior of networks. Hence we do not address here the evolutionary trajectories of threshold loci for the feedforward network. These can be found in the Supplementary Material (Text S1 and Figure S1).

### Feedforward network

#### Optimal worker distribution 1∶1

**Figure 2:**
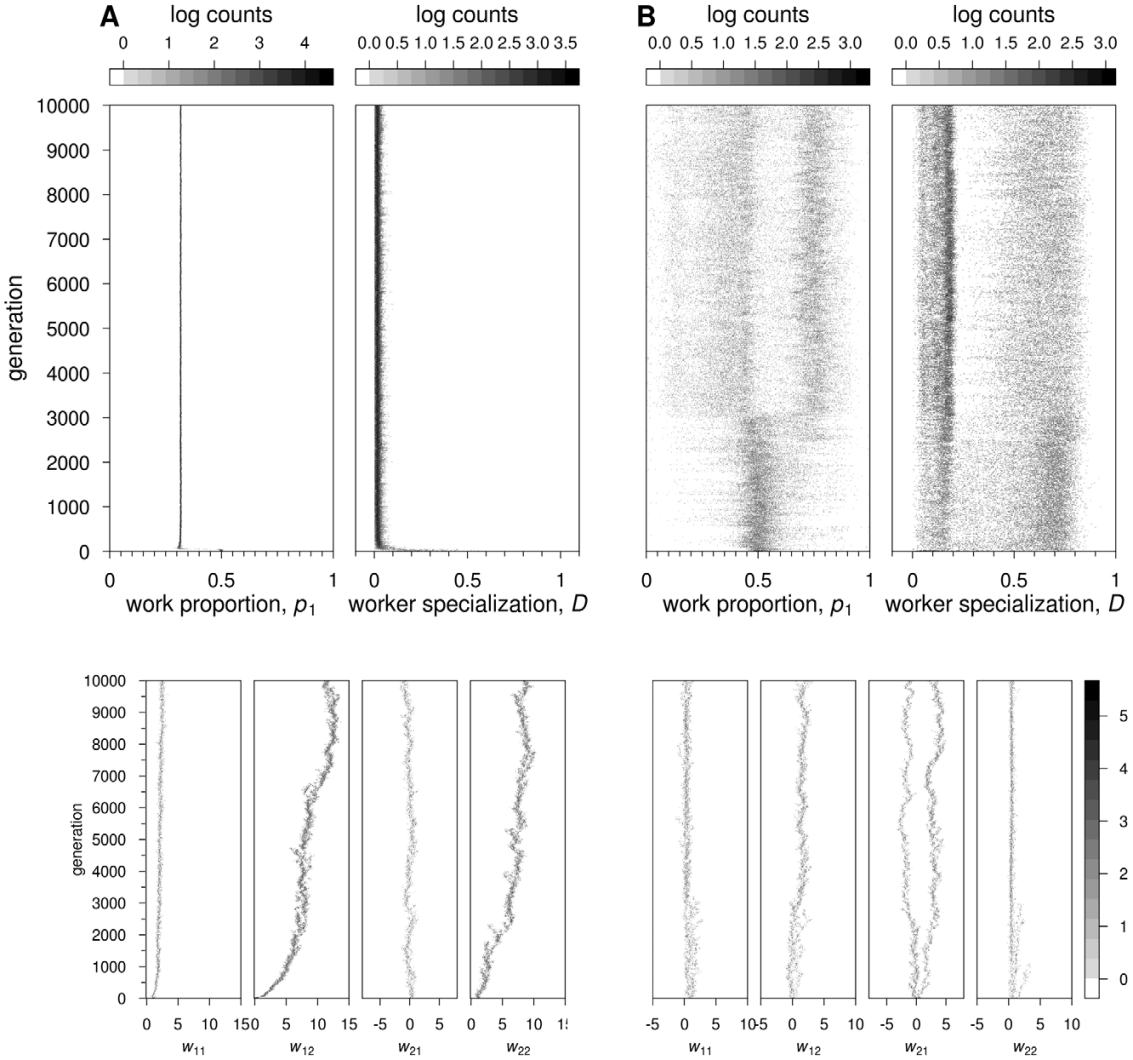
Feedforward neural networks: Evolutionary dynamics of two representative simulations, for 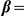
½ **and** 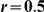 **.** Grey scales indicate log counts of colonies with the corresponding value of 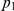, 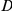 (scales on top of the respective graphs) and connection weights (scale on the bottom right-hand side). (A) No switching costs (*c* = 0). Top graphs: 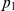 decreases to approximately 0.3. Worker specialization remains at zero. Bottom graphs: incoming connection weights at output node 2 evolve to strong positive values, whereas incoming connections weights at output node 1 evolve to weak positive values (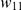) or oscillate around zero (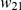). (B) With switching costs (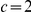). Top graphs: the distribution of workers over tasks and the degree of worker specialisation are both highly variable across colonies. At the end of the simulation, 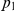 and 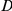 are both bimodally distributed. Bottom graphs: one of the connection weights (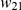) branches, one branch having positive values and the other, negative values. All other connections show weak positive values or remain very close to zero, all being relatively homogeneous in the population.

When 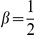, both tasks are equally needed, and a 1∶1 distribution of workers over tasks would be optimal (see (3)). It is therefore somewhat surprising that, in the absence of switching costs, all replicate populations evolved a work distribution where one of the tasks was performed three times more often than the other (Figure 2A, top panel). From here on we refer to the task performed most often as the “preferred task”. Which task was preferred varied among replicate populations, but within a population all colonies preferred the same task. Variation among colonies in fitness values was small; all colonies reached approximately 94% of the maximum fitness (see Figure S3). Higher fitness values could not be achieved due to the deviation from a 1∶1 task distribution.

Typically in our simulations, both ‘incoming’ connection weights of one of the two output neurons (the neuron corresponding to the preferred task) became positive over evolutionary time (Figure 2A, bottom panel). As for the incoming connections of the other output neuron (corresponding to the non-preferred task), the direct connection (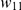 in the example simulation of Figure 2A) became positive, while the cross-connection (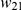, in Figure 2A) typically became weak, oscillating between positive and negative values. In all simulations, the strongest positive connection was between the stimulus input neuron of the non-preferred task to the output neuron of the preferred task (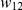, in Figure 2A). Hence, individuals use the stimulus for one task (their non-preferred task) to motivate them for performing the other task (their preferred one). As a consequence, they continue performing their preferred task, even if the stimulus level of this task has become very low (Figure S2). For this parameter combination (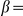
½, 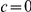), the degree of recombination had no effect on the outcome of the simulations (Figure S4A).

In the presence of switching costs, the results are considerably different. When switching costs were low (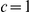), worker specialization only evolved in the absence of recombination (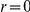), with 61.4±7.2% of the colonies (mean ± SD) evolving values of 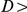
0.5. When *c* = 2, worker specialization also evolved in the presence of recombination (Figure 2B). Here 35.6±8.2% of the colonies showed 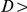
0.5. In all simulations with 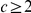 there was a clear (but weak) positive relationship between colony fitness and the degree of worker specialization within the colony; colonies with high mean specialization have a fitness advantage of approximately 20% over non-specialized colonies (Figure S3).

The bias in favor of one of the tasks that was observed in the absence of switching costs was much less pronounced or even absent in the presence of such costs. For 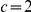, initially most colonies show a work distribution close to 1∶1 (Figure 2B, top panel). After about 3500 generations, a new pattern arises, with part of the colonies having a pronounced bias toward task 1, while the other colonies have a bias toward task 2. The simulation shown is representative for higher switching costs (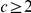), but to a certain extent the outcome depends on the detailed assumptions. If, for example, recombination was not allowed in the simulation of Figure 2B (i.e., 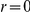), three different types of colonies evolved (with 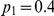, 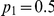 and 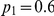, respectively; see Text S1 and Figure S4B).

**Figure 3:**
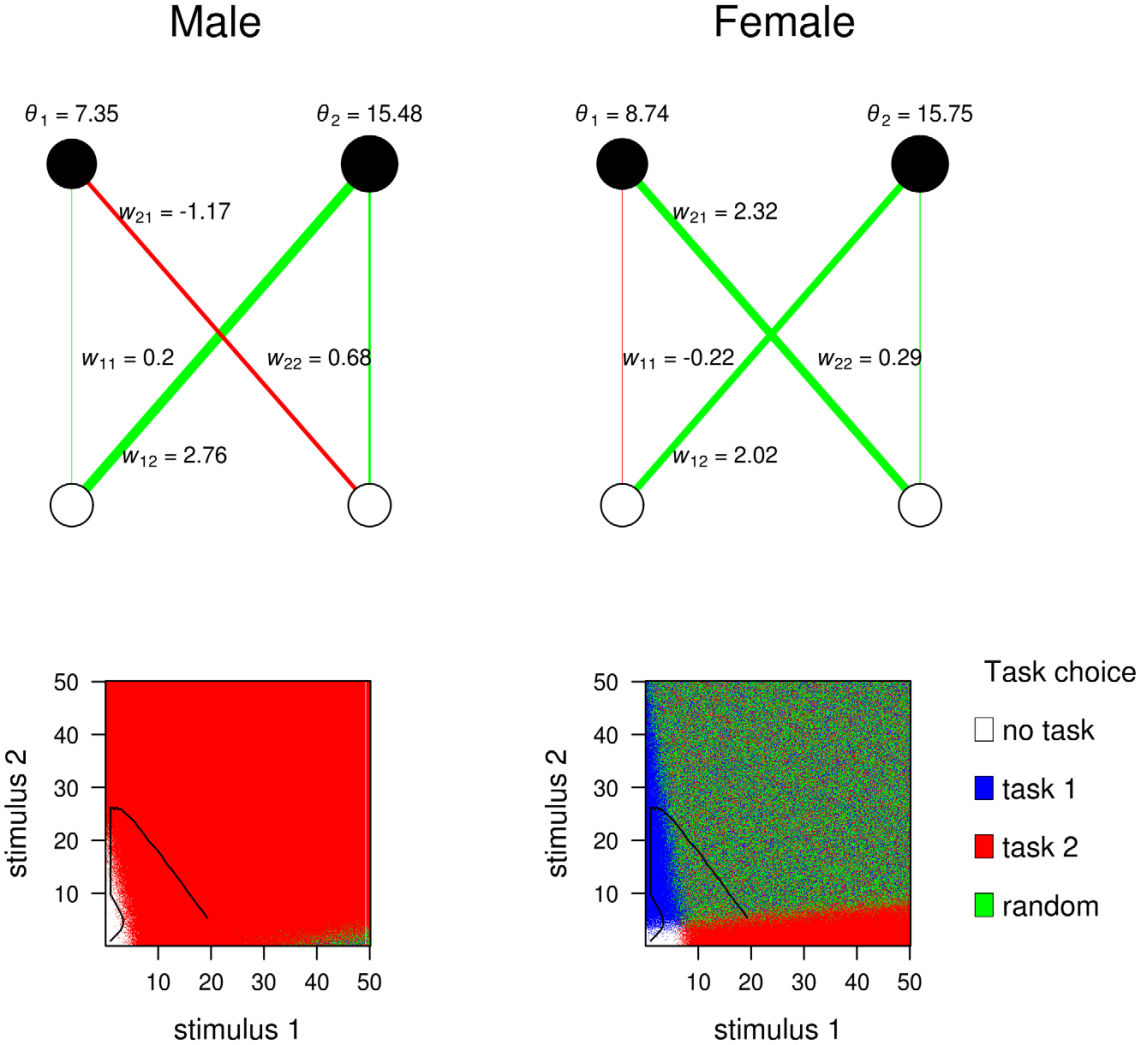
Evolved feedforward neural networks of a highly specialized colony. In specialized colonies, the networks of the two parents (arbitrarily labelled ‘male’ and ‘female’) differ from each other in a systematic way. Top panels: for each parent, the evolved values of the connection weights and thresholds of the network are shown. Bottom panels: the stimulus-response characteristics of each network type are shown. For each combination of stimuli, the bottom graphs show whether the network is motivated to perform only task 1 (blue), only task 2 (red), both tasks (green; in this case, one task is chosen at random), or none (white). The trajectory of stimulus values from the start to the end of the work phase, in the last generation of the evolutionary simulation, is indicated in black. Starting values were 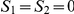. Other parameter values as in Figure 2B.

The neuronal connection weights linking input neurons to the corresponding output neurons (*i.e.*, 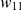 and 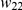) tended to evolve positive values, between 0 and 4 (Figure 2B, bottom panel). One of the cross-connections (*i.e.*, 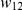 or 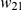) showed evolutionary branching [28], that is, polymorphism evolved from an initially monomorphic state. Figure 2B is representative in that 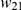 branches into a bimodal distribution, with one branch becoming negative and the other positive. When such branching occurs, two distinctly different types of networks coexist in the population (Figure 3, top panel). This is crucial for worker specialization: a high degree of specialization only occurred in colonies where the two parents differed in the sign of one of their cross-connection weights. From Figure 3 we can deduce how specialization occurs in a colony with dissimilar parents. The key difference between the parents' networks is the genotypic value of 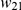, which determines that one parent (arbitrarily labelled ‘male’) is a specialist for task 2, while the ‘female’ shows a large area of the stimulus space where both tasks are activated and where accordingly one of the two tasks is chosen at random (Figure 3, bottom panel). The workers produced by these parents will be divided among those two phenotypes. Stimulus increase initially occurs for both tasks, until the stimuli levels reach a region where individuals with a positive *w*_21_ will perform task 1. As a consequence, only stimulus 2 will keep increasing, until an area is reached where individuals with a negative 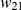 will start performing task 2. The decreasing stimulus of task 2 means that fewer workers will do task 1, because the main motivating force to do task 1 is the positive 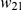. Hence, stimulus for task 1 will also increase. Individuals are then in an area of the stimulus space where half of them will work randomly on either task, while the other half will only perform task 2.

For 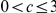, branching occurred at only one of the cross-connections, while for 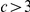 both cross-connections branched in some of the simulations.

In the absence of recombination, evolution leads to a higher degree of worker specialization (Figure S4B). Evolutionary branching occurs now for all the connection weights and for the thresholds as well. The area in stimulus space where networks choose both tasks is much smaller in the absence of recombination (Figure S5), leading to more pronounced differences between workers and, hence, more specialization. Branching of more loci means that networks will be more differentiated than seen previously for cases with recombination. .

#### Optimal worker distribution 3∶1

**Figure 4:**
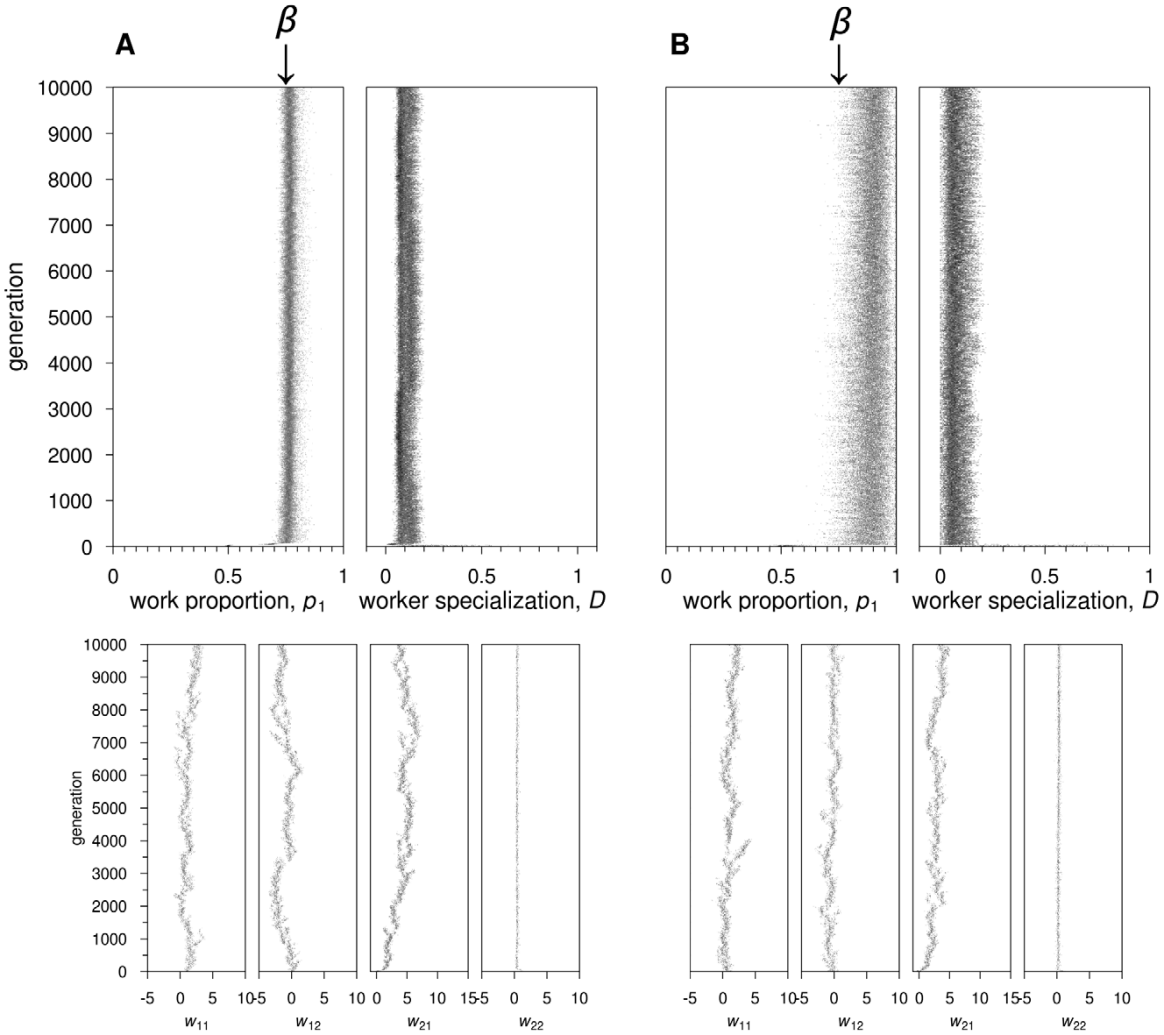
Feedforward neural networks: Evolutionary dynamics for two representative simulations, where 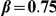
 **and** 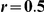 **.** The same graphic conventions as in Figure 2 are followed. (A) 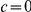. Top graphs: 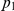 increases to the optimal value 0.75; specialization remains low. Bottom graphs: connection weights incoming at output node 1 become positive (strongest connection weight being 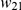); connection weights incoming at output node 2 become negative (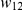) or positive, but very close to zero (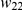). (B) 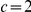. Top graphs: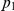 increases to values above 0.75; 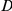 remains low. Bottom graphs: similar to when 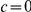, but 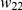 is closer to zero.

In view of eq. (3), when 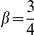, the optimal worker distribution over tasks is 3∶1, with task 1 being performed 3 times more often than task 2 (i.e. 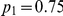). Populations indeed evolved a worker distribution approaching this value (Figure 4A, top panel). In absence of switching costs, task 1 was performed 76.7±0.42% of the time (mean ± SD across all replicate populations, for 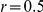). All colonies attained more than 99% of maximum fitness, with a few colonies achieving the maximum (Figure S3).

A general pattern in the evolution of connection weights was the strengthening of the cross-connection 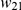 and the disappearance of connection 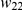 (as in Figure 4A, bottom panel). This explains the observed increase in performance of task 1. The cross-connections once more play an important role; since the strongest incentive to do task 1 comes from the stimulus of task 2, this allows workers to keep doing task 1 even if the stimulus for that particular task is depleted.

Worker specialization did evolve, but only in the absence of recombination (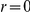). Even then, specialization levels of 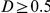 were only obtained for a larger number of colonies when switching costs were high (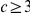). When worker specialization did not evolve (as in Figure 4B, top panel), colonies evolved work distributions even more biased than 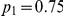. When the work distribution is that strongly biased, the probability to stick to the previous task (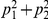) is high even if tasks are taken on at random. Hence, by evolving a work distribution with more than 80% of the work devoted to task 1the number of switches decreases, thus allowing colonies to avoid switching costs even in the absence of worker specialization. In this case, connection 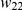 reached lower values than for the simulations without switching costs (Figure 4B, bottom panel).

### Recurrent network: self-feedback

We tested the behavior of a more complex network, where the activation energy of an output neuron could have a feedback on the activation energy at the next time step (Figure 1C). The self-feedback connections were allowed to co-evolve with the rest of the network. We ran ten replicate simulations for all the parameter combinations tested above.

#### Optimal worker distribution 1∶1

**Figure 5:**
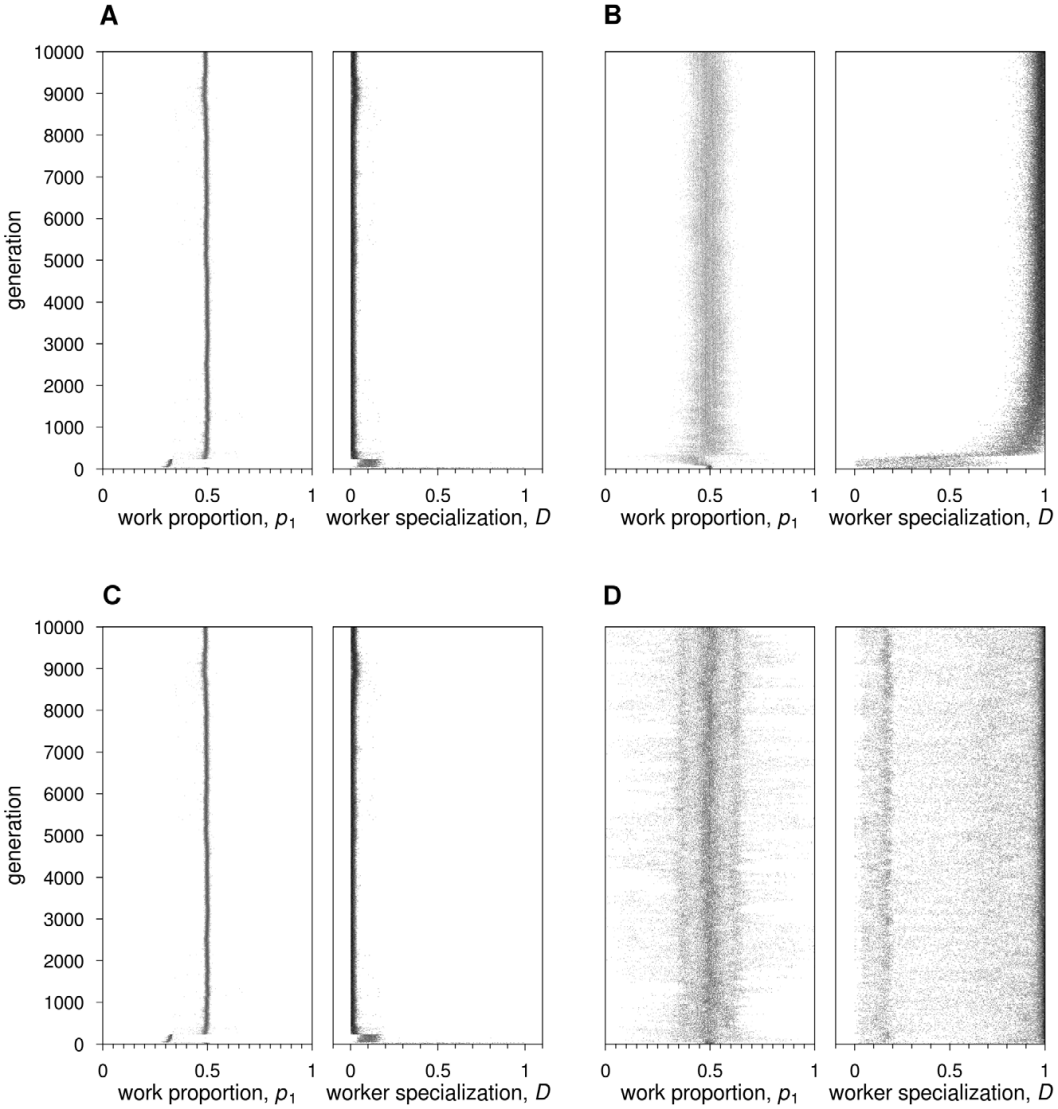
Recurrent neural networks: Evolutionary dynamics of the proportion of time spent on task 1, 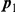
 **, and the degree of worker specialisation,** 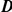 **.** Two representative simulations are shown for 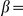
½. (AB) 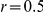. In (A), switching costs are absent: 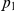 quickly reaches the optimal value 0.5; worker specialization does not evolve. In (B), 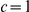: 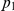 becomes more variable, but still approximates the optimal value 0.5; 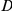 rapidly increases to its maximal value 1, for all colonies in the population. (CD) 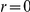. In (C), switching costs are absent: the evolutionary dynamics is as in (A). In (D), 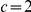: not all colonies can evolve worker specialization, and 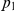 is also more variable across colonies.

In contrast to the results of the feedforward network, the optimal worker distribution 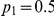 was now realized in a high proportion of colonies (*e.g.*, figs. 5AB). However, this proportion decreased with increasing switching costs. For 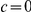, the proportion of colonies with 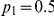 was 99.9±0.3% when 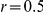 and 100% when 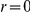. For 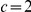, this proportion was at 76.5±4.1% when 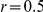 and 46.7±7.6% when 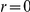 (mean ± SD number of colonies across replicates).

When 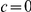, all colonies in all replicate simulations achieved the maximum possible fitness, indicating that all workers are active all the time (Figure S6). Workers switched randomly between tasks (*D* = 0 for all colonies, Figure 5A). This was achieved by evolving positive self-feedback connections allowing workers to continue working even in the absence of an external stimulus for a task. Connection weights from stimuli input neurons to output neurons were also positive (Figure S7).

Worker specialization evolved already for low switching costs (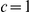), but the behavior shown by colonies, for all 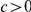, differs considerably in the simulations in the presence or absence of recombination. In the presence of recombination, all colonies within a population reached a high value of *D* (Figure 5B). In the absence of recombination, populations typically consisted of colonies with low 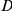 and colonies with high 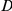 (Figure 5D). For *c* = 1, for example, 25±7% of the colonies (mean ± SD across replicates) had 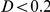, while 66±7% of colonies showed 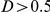.

**Figure 6:**
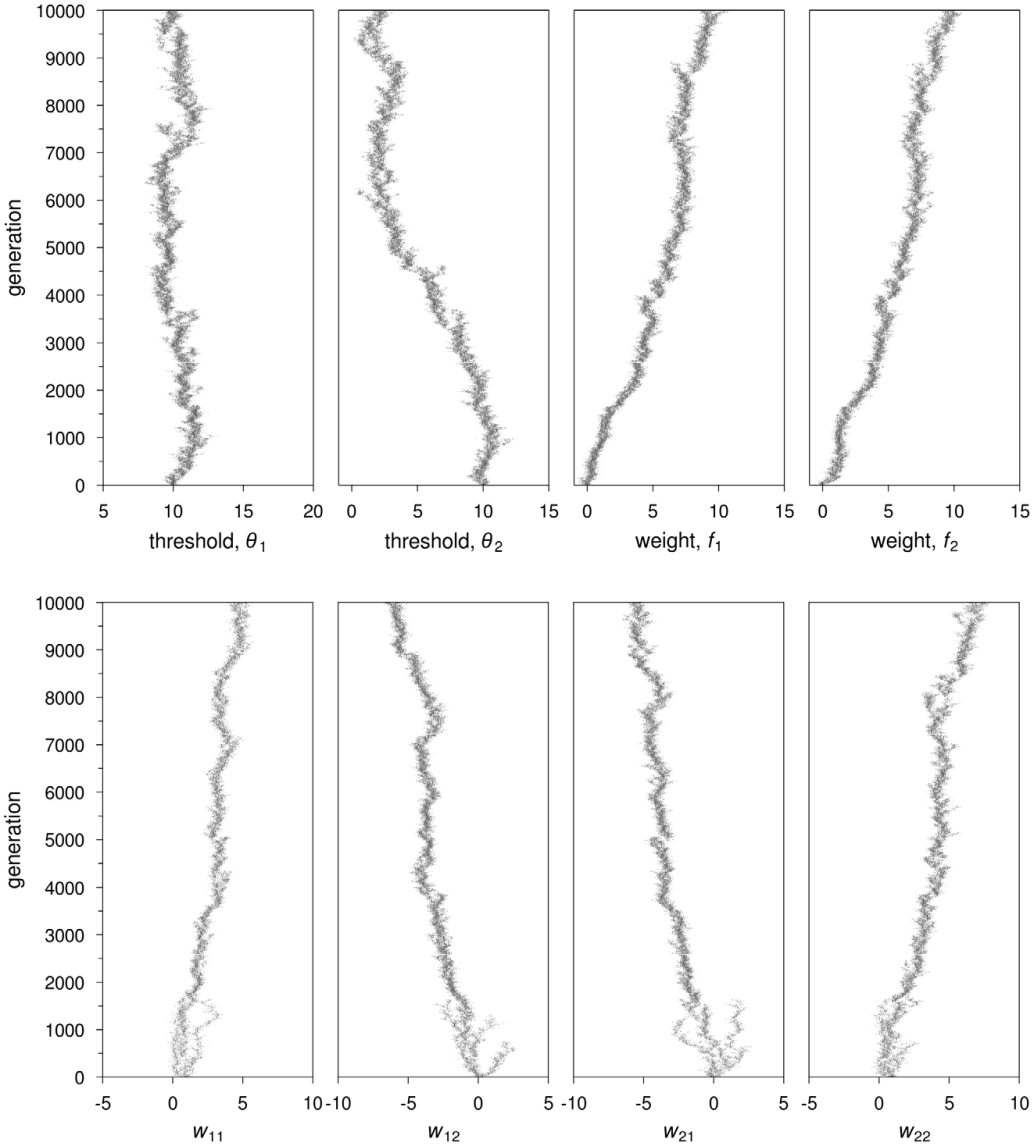
Recurrent neural networks: Evolutionary trajectories of network parameters leading to experience-based specialization. A simulation is shown in which all colonies in the population evolve high degree of division of labor. Parameter values are: 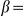
½, 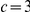, 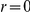. The self-feedback connection weights, 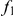 and 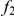 (third and fourth graph on the top panel), increase over generations, a pattern which is found across simulations showing the same worker specialization patterns. Also representative is the pattern encountered in the other connection weights (bottom panel) is the evolution of negative values in cross-connection weights (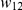 and 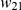) and positive values in the connection weights between the task stimulus and respective output node (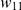 and 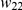).

In the simulations where all colonies exhibited a high level of worker specialization, self-feedback connections evolved very high positive values (as in Figure 6, top panel). The connection weights from task stimulus to corresponding output neuron (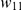 and 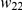) evolved to positive values, while cross-connection weights (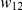 and 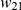) evolved to negative values (as in Figure 6, bottom panel). In these simulations, the evolved strategy leading to division of labor uses the strong self-feedback connections, accompanied by negative cross-connection weights, to create differentiation between individuals. Since individuals from the beginning perceive different levels of stimuli, differences in activation energy will occur and will be amplified in subsequent time steps, creating consistent differences among individuals. Hence division of labor is achieved by experience-based specialization.

**Figure 7:**
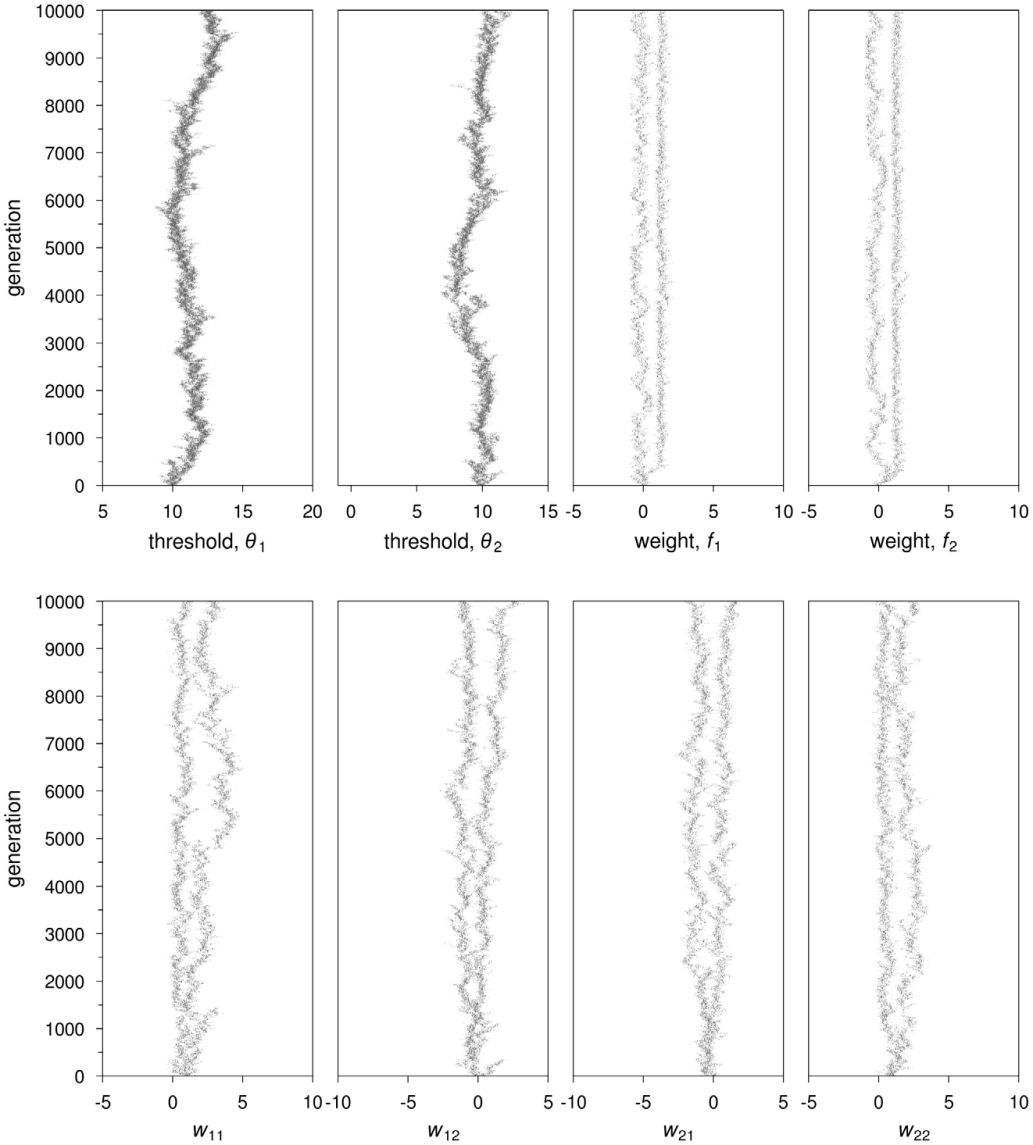
Recurrent neural networks: Evolutionary trajectories of network parameters leading to genetically-determined specialization. A simulation is shown in which only half of the colonies in the population evolve a high degree of specialization. Parameter values as in figure 6. All connection weights undergo evolutionary branching. The self-feedback (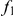 and 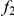) and crossed connection weights (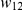 and 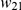) show one branch with negative values and the other with positive values. The other connection weights show one branch close to zero and the other larger, positive values.

In the simulations where colonies differ in their degree of worker specialization, neuronal connections (including self-feedback connections) show evolutionary branching, with one branch showing positive values and the other branch negative values or values close to zero (Figure 7). In this case, evolutionary branching allows for the co-existence of different genetically determined specialists, as seen previously for the simpler feedforward architecture.

#### Optimal worker distribution 3∶1

**Figure 8:**
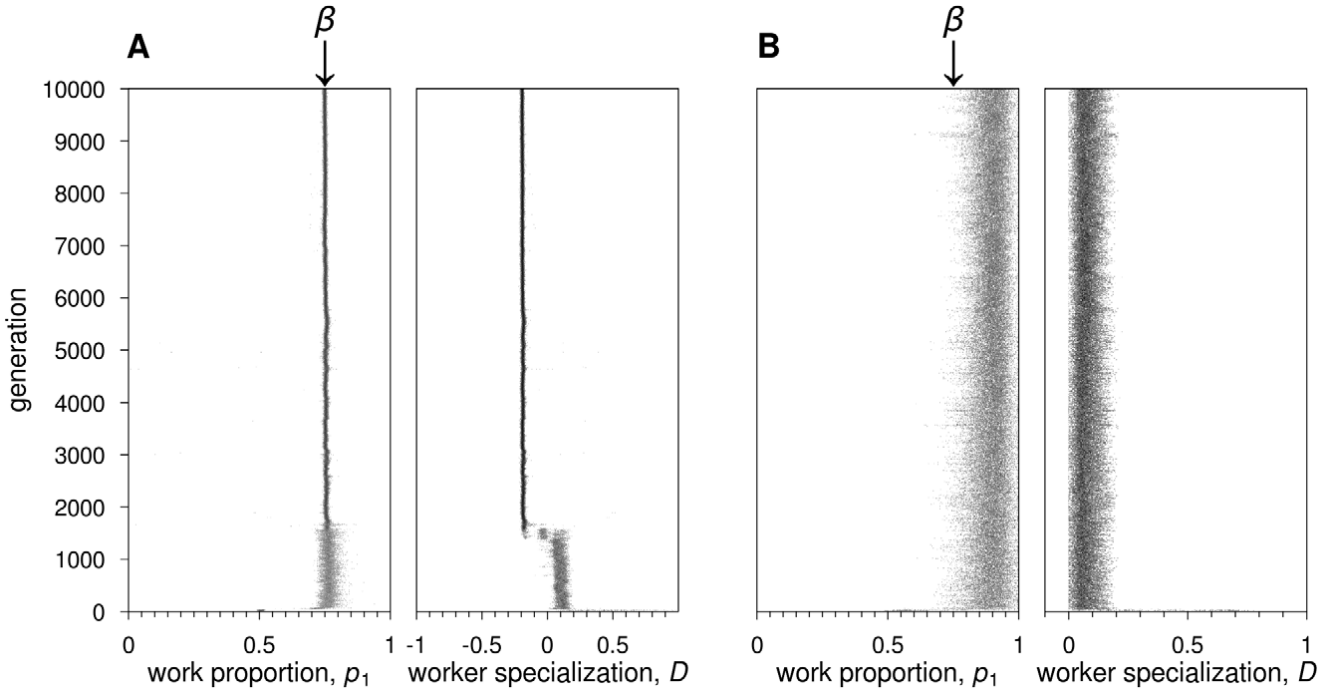
Recurrent neural networks: Evolutionary dynamics of work distribution 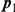
 **and worker specialisation** 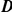 **for** 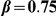 **and** 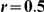 **.** (A) 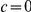. 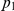 quickly reaches the optimal value 0.75. 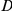 evolves to negative values, indicating that individuals switch tasks more often than by chance. (B) 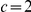. 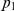 increases to values above 0.75; worker specialization does not evolve.

In the absence of switching costs, the mean 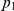 calculated across replicates was 0.75 and, hence, corresponding to the optimal value for fitness (Figure 8A). Interestingly, worker specialization was negative (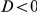) in all colonies in 19 out of 20 simulations (encompassing both simulations where recombination is present as well as where it is absent). In other words, individuals switched more often between tasks than expected by chance.

Worker specialization never evolved for 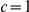. For 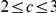, specialization only evolved in the absence of recombination. These results are shown in the Supplementary Material (Text S2, Figures S8, S9, S10). For 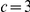, in two of the replicates, all colonies show high levels of specialization, accompanied by the optimal worker distribution (Figure S8A). In these particular replicates the self-feedback connections became strongly positive (Figure S9). In all other replicates only about half the colonies showed 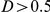, while the other half had no specialization (Figure S8B). The distribution of workers over tasks was highly variable, with very few colonies actually achieving 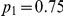. The networks in these populations showed evolutionary branching of self-feedback connections (Figure S10).

For higher switching costs (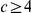), worker specialization could evolve in the presence of recombination, but only in three replicates out of 20, in the evolutionary time considered (results not shown). In these replicates, all colonies combined high levels of specialization and a work distribution very close to the optimal value of 0.75. Worker specialization was again achieved through two different types of networks; one where evolutionary branching occurs in key neuronal connections (particularly self-feedback connections), and the other through evolution of strong positive self-feedback connections (not shown). The first network type leads to a population where only half the colonies have specialized workers, and the correct work proportion is hardly achieved; the second network type leads to a population where all colonies have a high level of specialization and the optimal work proportion.

## Discussion

Here we studied whether and how two different neural network architectures enable the evolution of self-organized division of labor and adaptive task ratios. Our results are summarized in table 1.

**Table 1:** Work proportion and degree of division of labor obtained under different behavioral architectures.

		*r* = 0			*r* = 0.5			
Work ratio	Result	*c* = 0	*c* = 1	*c*≥2	*c* = 0	*c* = 1	*c*≥2	Model
***β*** ** = 0.5**	***p*** **_1_ = ** ***β***	Y	Y	Y	Y	Y	Y	RT
		N	P	P	N	N	P	FFN
		Y	P	P	Y	Y	P	RN
	***D*** **>0.5**	N	P	P	N	N	P	RT
		N	P	P	N	N	P	FFN
		N	P	P^1^	N	Y	Y	RN
***β*** ** = 0.75**	***p*** **_1_ = ** ***β***	N	N	N	N	N	N	RT
		Y	P	N	Y	P	N^2^	FFN
		Y	P	P^3^	Y	P	N^3^	RN
	***D*** **>0.5**	N	N	P	N	N	P	RT
		N	N	P^1^	N	N	N	FFN
		N^4^	N	P^3^	N^4^	N	N^3^	RN

1 for *c*≥3.

2 except small percentage (<3%) when *c* = 2.

3 with exception of few simulations, where all colonies obtain the result.

4 D<0.

Overview of results obtained for three different behavioral architectures: RT – response threshold model (A. Duarte, I. Pen, L. Keller and F.J. Weissing, subm.); FFN – feedforward neural network; RNN – recurrent neural network. Parameter combinations are indicated in the first column and first two rows. The second column indicates the result we look for: “
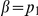
” corresponds to the achievement of the optimal work ratio; “
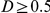
” corresponds to the evolution of worker specialization. In the central columns, for different levels of switching costs, *c*, we indicate if such results were obtained. “Y” indicates it was satisfied in all replicate simulations; “N” indicates that the result was not obtained, in the majority of simulations; “P” indicates that, in the majority of simulations, a fraction of the colonies within the population obtained the result.

With a feedforward network (table 1), worker specialization evolved more easily (i.e. at lower switching costs) in the absence of recombination. In the absence of recombination the connection weights can co-evolve as a tightly linked block of genes, making it easier to evolve specific combinations of connection weights favoring specialization. Recombination pushes populations into a solution where only one connection weight locus branches, the rest of the network being relatively homogeneous in the population. This allows worker specialization to occur, but to a lesser extent than in the absence of recombination, because at least one of the parent networks in a specialized colony behaves as a generalist for a large range of stimulus combinations. A large percentage of colonies showed no worker specialization, hence, no division of labor. This is because random mating allows for couples with similar genotypes to produce colonies where workers are too similar and therefore division of labor cannot emerge.

Previous work on the response threshold model (A. Duarte, I. Pen, L. Keller and F. J. Weissing, subm.) showed that the work ratio could not easily deviate from 1∶1, even if a biased work ratio was optimal. In contrast, in the case of the feedforward network, the work ratio was always biased for one of the tasks, even when a symmetric work ratio was optimal (table 1). Owing to selection for minimizing idleness, the evolved networks maximized the amount of work done by using the stimulus from one of the tasks to stimulate workers to perform the other task. In this way, one of the tasks was performed in excess (the ‘preferred’ task), even when its associated stimulus had been depleted. Although this may seem counter-intuitive, it represents an advantage over networks that attempt to maximize both tasks, because these networks would be limited to the work strictly necessary to reduce stimuli to zero. When 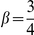, the optimal work ratio was achieved, but only in the absence of switching costs. When switching costs were present, the most common evolved strategy was to increase the proportion of work for task 1 in order to minimize switching among tasks.

Some of the limitations of the simple feedforward network were eliminated in the slightly more complex architecture of the recurrent network, where previous activation energies feed back on current activation energies. Worker specialization evolved at low switching costs, now both in the presence and absence of recombination (table 1), at least for 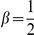. Interestingly, the presence of recombination favored an outcome where all colonies showed a high degree of specialization. In these populations, specialization does not depend on the presence of two complementary networks in the parents of a colony (as in figure 3), but on a strengthening of the self-feedback connections. This allows for initial differences between individuals in stimulus perception to be amplified in subsequent time steps and leads to behavioral differentiation through reinforcement of previous experiences. In the presence of recombination, this strategy prevails. However, when no recombination occurs, evolutionary branching of connection weights is still the prevalent strategy through which worker specialization evolves. Why is the experience-based strategy not observed in all simulations? A likely reason is that to reach this strategy, the values of neural connections must first pass through values where, in the absence of recombination, evolutionary branching is more advantageous. Hence, the evolutionary outcome is dependent on initial conditions. We confirmed this by running simulations where the self-feedback connections were initialized at higher values (e.g., 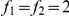); in this case all populations evolved the experience-based strategy rather than evolutionary branching (results not shown). The evolution of an experience-based strategy is affected by stochastic effects at the moment that the population passes the “branching point”, namely on the direction and magnitude of genetic variation, that may lead to local fitness optima. The two strategies may thus represent alternative stable states. The mean population fitness of the genetic specialization (evolutionary branching) is noticeably lower than the mean population fitness of the experience-based strategy (Figure S7).

The recurrent network also allowed for the optimal work ratio to be reached in most cases, at least by part of the population (Table 1), even in the presence of switching costs. When 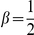, the self-feedback connections allow the continuous activation of both tasks, stimulating individuals that had previously done a task to do it again, even in the absence of the corresponding task stimulus. With this architecture it is also harder to attain the optimal work ratio when 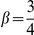 and switching costs are considered, and only few replicate populations show both 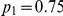 and high degree of worker specialization.

The recurrent network has similarities with the reinforced threshold model, in which individual thresholds are lowered after the performance of the respective tasks and increased when the tasks are not performed [9], [29]. In both models, initial differences in experience lead to consistent behavioral differentiation, thus bypassing the need of specific genetic combinations for the emergence of task specialization. However, in terms of the distribution of workers over tasks, the reinforced threshold model suffers from the same limitations as the fixed threshold model, with worker distribution being mainly dependent on the parameters of stimulus dynamics (A. Duarte, T. Janzen, F.J. Weissing and I. Pen, in prep.).

Our results highlight the importance of considering asymmetries in models of division of labor. In the evolutionary response threshold model by A. Duarte, I. Pen, L. Keller and F. J. Weissing (subm.), we show that a biased 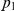-value cannot be obtained through the evolution of thresholds. To achieve a biased 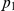-value in this model, asymmetry must be present in the environment (e.g. in the values of task-associated stimuli [8]) to which the response-threshold mechanism then responds. However, in reality, asymmetries in the work distribution might also arise from the ability of individuals to perceive and prioritize tasks differently. Here we show that, for both types of networks studied, it is not easy to evolve strict worker specialization together with an asymmetric distribution of workers over tasks. A major difficulty is that in case of genetically determined specialization the work proportion is dependent, to a large extent, on the proportions of different specialists in each colony. Since we only consider single-mated foundresses, colonies in our model show either equal proportions of the two specialist strategies or only one of the specialist strategies. Evolving experience-based specialization enables an asymmetric work distribution and division of labor (although at a lower degree of worker specialization than under symmetric conditions, and only in the absence of recombination), yet the trajectory towards this strategy is subject to stochastic effects that may diverge evolution towards genetically determined specialization or towards an increase of performance of the most needed task beyond its optimal level.

The observed difficulty in favoring a specific work ratio under switching costs indicates that the simple behavioral architectures investigated are limited in the ability to evolve efficient solutions to complex optimization problems. In the presence of switching costs, it is important for colonies to maximize worker specialization, while at the same time minimizing the number of idle workers *and* optimizing the work ratio. The behavioral architectures considered thus far were only able to evolve sub-optimal solutions to this multi-faceted problem.

Modelling the evolution of behavioral mechanisms by means of artificial neural networks presents several advantages when compared to *a priori* chosen behavioral architectures such as a response threshold mechanism. First, mechanisms potentially leading to self-organized division of labor are not built into the model, but must emerge from the model. Second, evolving neural networks transcend some limitation of the human mind. When asked to design plausible mechanisms, the imagination of most modellers is limited to simple and intuitive mechanisms (like a response-threshold mechanism) that our mind can easily envisage. For example, it is unlikely that one would envisage a mechanism where a task-associated stimulus does not stimulate the performance of its corresponding task, but of a different one, as it occurs in the feedforward network. By using an independent modelling setup, we can get an idea whether, and to what extent, the results based on the more standard implementations are robust. In our case, the simple feedforward network is too constrained to achieve worker specialization and an appropriate distribution of workers over tasks. By adding a simple elemental feedback the resulting recurrent network had a much higher evolutionary potential. In future models we could consider the evolution of the network's topology, e.g. by allowing the addition and elimination of neurons and connections to an existing network through mutation [24].

The simple feed-forward neural network was constrained by a problem already present with the response threshold mechanism: to get specialization at the colony level, the coexistence of two specialist genotypes is necessary. Random mating and recombination played an important role in the evolutionary outcome. In general we observed that recombination made it more difficult for genetic specialization to evolve. With recombination, evolutionary branching at multiple loci occurred only rarely, at very high switching costs. This is in accordance with the argument that, in constant environments, recombination may destroy favorable allelic combinations [30], [31]. Our model suggests that in systems where strong genetic task determination and high recombination rates exist, multiple mating would be favored, in order to increase the chance that workers have favorable allelic combinations. This is in accordance to what we observe in honeybees [32], [33]. Under the recurrent network architecture, recombination may also play a beneficial role by creating more genetic variation in the self-feedback connections, which could favor division of labor emerging through the experience-based strategy.

The purpose of our approach was not to represent the behavioral architecture of real organisms, but to present a conceptual model that could shed some light on the role of architectural constraints in the evolution of self-organized division of labor. A limitation of this approach is that the larger the network, the more difficult it is to draw conclusions that are biologically relevant. We have implemented two very simple networks, and yet already have six to eight evolvable parameters. We were able to understand the interaction of the networks with the environment and pinpoint the key connections that allowed for specific behaviors, but this may not be possible for more complex architectures.

The fitness function used (eq. 2) favored the minimization of idleness. Although it is not unrealistic to assume that more work will translate to higher colony productivity, in reality social insect colonies contain a large proportion of idle workers [34]–[36]. Examples of circumstances that would allow the presence of idle workers include environmental perturbations that require quick recruitment of “stand-by” workers, advantage of energy-saving strategies under poor resource conditions, and selective neutrality of “incompetent” workers due to highly redundant organization of work [36] (and references therein). As stressed before, here we present a conceptual model for the effect of behavioral architectures in division of labor, and necessarily simplify certain assumptions. A more realistic version of our model would treat fitness as the number of offspring produced by a colony, and explicitly consider the nature of the different tasks (e.g. foraging and brood care).

Division of labor is a broad topic, with many aspects that were outside the scope of this study. Previous theoretical work has focused on the evolution of differentiated multicellularity, the evolution of germ and soma in multicellular organisms, and the effect of developmental plasticity in gene expression as a cause of individual differentiation [37]–[40]. Here we focused on the evolution of behavioral task specialization in groups where reproductive altruism (analogous to germ-soma differentiation) has already evolved, an assumption which is in line with a recent comparative analysis of the evolutionary history of division of labor [41]. We did not consider the role of developmental plasticity, although this plays an important role in the differentiation of morphological castes [42]. Underlying the different questions concerning division of labor, however, is a problem of functional optimization: Organisms can increase their reproductive success if they perform different tasks efficiently. Dividing tasks among lower-level units within the organism or colony (often referred to as a superorganism) is a solution to the problem. What our model suggests is that the particular behavioral rules through which task specialization arises may impact the evolutionary outcome.

## Supporting Information

Figure S1Evolutionary trajectories of thresholds for four example simulations differing in the switching costs and the optimal work proportion, 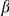. Graphic conventions follow figure 2 in main text. In all simulations, *r* = 0.5. (A) 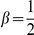, *c* = 0. (B) 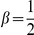, *c* = 2. (C) 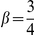, *c* = 0. (D) 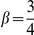, *c* = 2.(TIFF)

Figure S2Typical colony of the last generation of an evolutionary simulation (*c* = 0 and 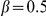). (A) Number of workers engaged in task 1 (black solid line) and task 2 (grey line) are indicated on the left-hand vertical axis, during time steps of the work phase. Degree of worker specialization, *D* (black dashed line), is indicated on the right-hand vertical axis. From the start, more workers engage in task 2 than task 1. Division Worker specialization close to zero throughout the simulation. (B) Stimulus for task 1 (black line) and task 2 (grey line) during the time steps of the work phase. Stimulus 1 remains at higher values, due to the fewer number of workers performing task 1.(EPS)

Figure S3Relationship between colony fitness and worker specialization at the end of evolutionary simulations of the feedforward network in the absence (AC) and presence (BD) of switching costs. For all colonies, fitness is represented as the fraction of the maximum possible fitness. In (AB), 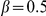. (A) 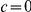, corresponding to fig. 2A in main text. All colonies achieve a high fitness; despite the fact that the evolved distribution of workers over tasks deviates substantially from the optimum value 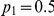 (see fig. 2 in the main text). As expected in absence of switching costs, there is no relationship between colony fitness and *D*. (B) 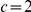, corresponding to fig. 2B in main text. Colony fitness increases with worker specialization, but even for large values of *D* colony fitness is substantially lower than in the absence of switching costs. In (CD), 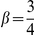. (C) 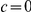, corresponding to fig. 4A in main text. All colonies achieve the highest possible fitness, because they are now able to achieve the optimal ratio among tasks (3∶1). As expected in absence of switching costs, there is no relation between fitness and *D*. (D) 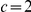, corresponding to fig. 4B in main text. Colonies do not reach high *D*, yet fitness changes with *D* in a non-monotonic way.(EPS)

Figure S4Evolutionary dynamics of two representative simulations of the evolution of a feedforward neural network, for 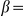
½ and 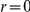. Figure follows graphic conventions of fig. 2 in the main text. (A) 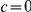. Top graphs: 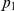 evolves to approximately 0.3. Worker specialization remains at zero. Bottom graphs: connection weights linked to output neuron 2 increase to positive values, the strongest being the cross-connection 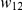. Direct connection weight 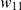 becomes positive, while the cross-connection 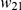 evolves to negative values. (B) 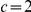. Top graphs: 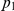 becomes more variable, with some colonies achieving the optimal value, 0.5, but most falling in one of two regions, one close to 0.4, the other close to 0.6. *D* rapidly evolves to a bimodal distribution with approximately 70% of the colonies having 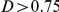 and approximately 30% having 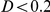. Bottom graphs: all connection weights suffer evolutionary branching. The cross-connections diverge the most, with one branch showing positive values and the other negative values.(TIFF)

Figure S5Evolved feedforward neural networks of the parents of a highly specialized colony in the simulation corresponding to fig. S4B (last generation). Top panels: evolved values of connection weights and thresholds are shown for each parent. Bottom graphs: the stimulus-response characteristics of each network are shown. For each combination of stimuli, the bottom graphs show whether the network is motivated to perform only task 1 (blue), only task 2 (red), both tasks (green; in this case, a task is chosen at random) or none (white). The black line indicates the trajectory of stimuli values during the work phase of the last generation of the evolutionary simulation. Starting values were 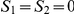.(TIF)

Figure S6Relationship between relative fitness and worker specialization, 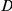, at the last generation of four representative simulations of the evolution of recurrent neural networks, for 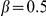. (A) 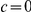, 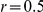: corresponding to fig. 5A in the main text. All colonies reach the highest possible fitness. (B) 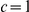, 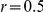: corresponding to fig. 5B in the main text. All colonies have high degree of worker specialization (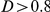). Colonies with the highest level of worker specialization are able to reach also the highest possible fitness. (C) 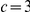, 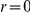: corresponding to fig. 6 in main text, one of the few cases in the absence of recombination where all colonies evolve worker specialization, and achieve maximum fitness. (D) Same parameter combination as (C), but depicting the more general pattern found in the absence of recombination and presence of switching costs (corresponding to fig. 7 in the main text). Only a portion of the colonies reach high worker specialization, which results in high variation in fitness among colonies.(EPS)

Figure S7Evolutionary trajectories of thresholds and connection weights of recurrent networks, in a simulation with 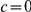, 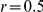 and 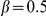, corresponding to Figure 5A in main text.(TIF)

Figure S8Evolutionary dynamics of two simulations of the evolution of a recurrent neural network, with self-feedback, for 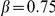, 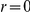 and 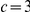. The simulations are examples of the two strategies that evolved in response to switching costs. (A) The less frequent outcome (2 out of 10 simulations), where all colonies show values of *p*_1_ close to 0.75, the optimal value, and most colonies show 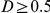, at the end of the considered evolutionary time. (B) The more frequent outcome, where approximately half the colonies showed 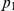 around 0.5 and 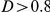, and the other half showed 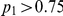 and 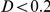.(TIFF)

Figure S9Evolutionary trajectories of thresholds and connection weights of recurrent networks, in a simulation with 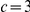, 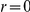 and 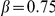, corresponding to fig. S7A. Top graphs: self-feedback connection weights evolve positive values, as in other simulations where all colonies showed high degree of worker specialization. Evolution of thresholds did not show a specific pattern across simulations, hence it plays a less important role in the outcome. Weights showed positive values for direct connections (with 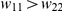) and 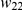) and negative values for cross-connections (with 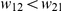), a pattern also representative for other simulations where all colonies evolved worker specialization.(TIFF)

Figure S10Evolutionary trajectories of thresholds and connection weights of recurrent networks, in a simulation with 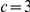, 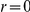 and 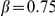, corresponding to fig. S7B. Top graphs: Self-feedback connection weights go through evolutionary branching, as in other simulations where only a portion of the colonies shows high degree of worker specialization. One branch has positive values, and the other negative values. Bottom graphs: weights are maintained at quite low values, oscillating around zero.(TIFF)

Text S1 Feedforward neural network.
(DOC)

Text S2 Recurrent neural networks, with self-feedback.
(DOC)
