# Supplementary figures and images for: Implications of Behavioral Architecture for the Evolution of Self-Organized Division of Labor
Source: PLoS Comput Biol. 2012 Mar 22;8(3):e1002430. doi: 10.1371/journal.pcbi.1002430 (PMC3310710; doi:10.1371/journal.pcbi.1002430)

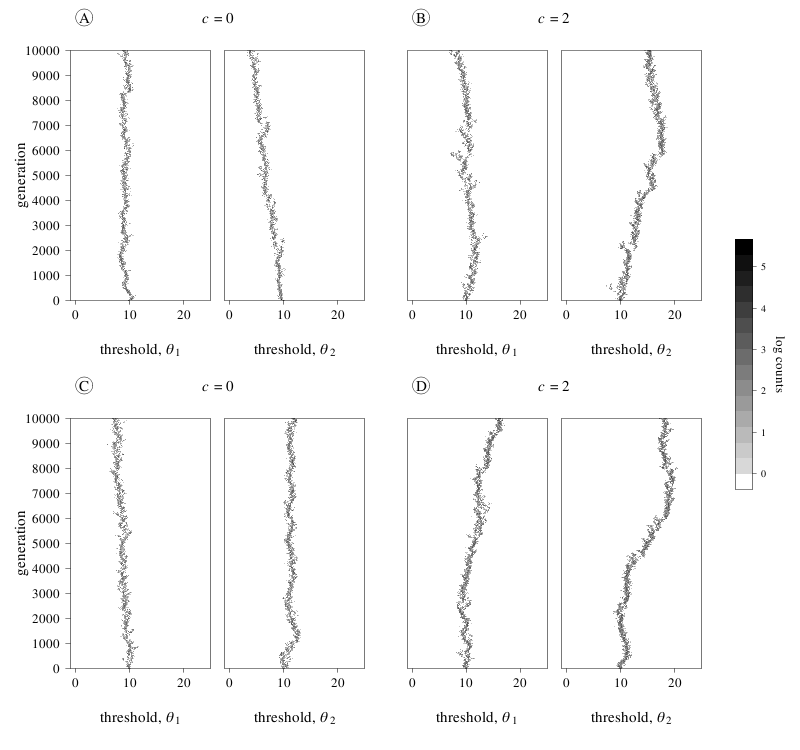

Supplement: Figure S1 — Evolutionary trajectories of thresholds for four example simulations differing in the switching costs and the optimal work proportion, . Graphic conventions follow figure 2 in main text. In all simulations, r = 0.5. (A) , c = 0. (B) , c = 2. (C) , c = 0. (D) , c = 2. (TIFF) [file pcbi.1002430.s001.tiff]

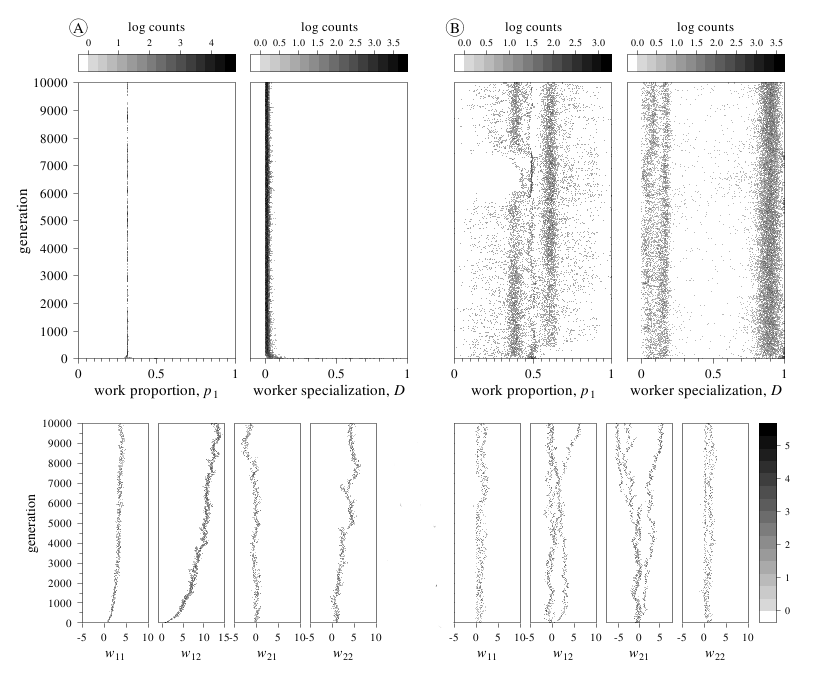

Supplement: Figure S4 — Evolutionary dynamics of two representative simulations of the evolution of a feedforward neural network, for ½ and . Figure follows graphic conventions of fig. 2 in the main text. (A) . Top graphs: evolves to approximately 0.3. Worker specialization remains at zero. Bottom graphs: connection weights linked to output neuron 2 increase to positive values, the strongest being the cross-connection . Direct connection weight becomes positive, while the cross-connection evolves to negative values. (B) . Top graphs: becomes more variable, with some colonies achieving the optimal value, 0.5, but most falling in one of two regions, one close to 0.4, the other close to 0.6. D rapidly evolves to a bimodal distribution with approximately 70% of the colonies having and approximately 30% having . Bottom graphs: all connection weights suffer evolutionary branching. The cross-connections diverge the most, with one branch showing positive values and the other negative values. (TIFF) [file pcbi.1002430.s004.tiff]

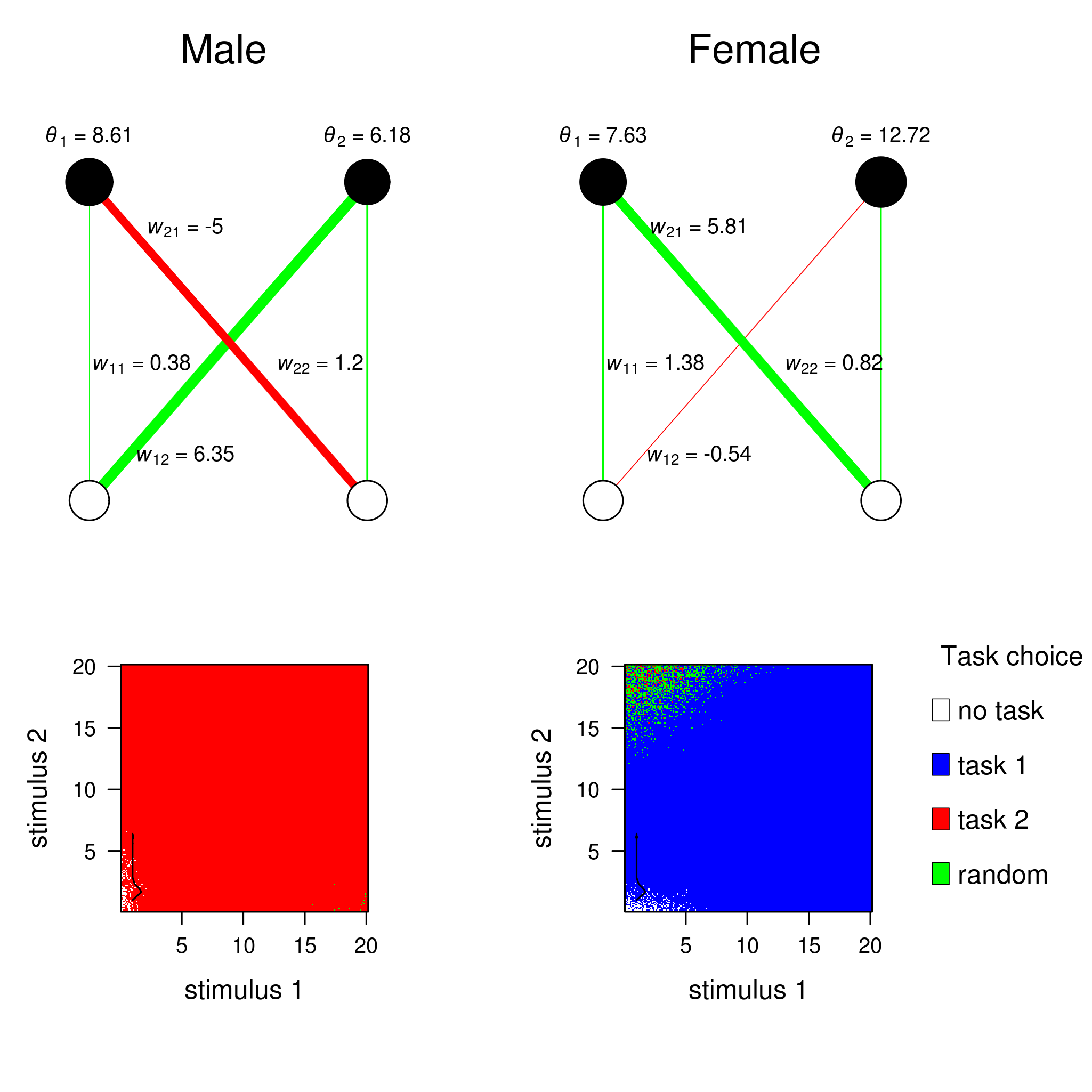

Supplement: Figure S5 — Evolved feedforward neural networks of the parents of a highly specialized colony in the simulation corresponding to fig. S4B (last generation). Top panels: evolved values of connection weights and thresholds are shown for each parent. Bottom graphs: the stimulus-response characteristics of each network are shown. For each combination of stimuli, the bottom graphs show whether the network is motivated to perform only task 1 (blue), only task 2 (red), both tasks (green; in this case, a task is chosen at random) or none (white). The black line indicates the trajectory of stimuli values during the work phase of the last generation of the evolutionary simulation. Starting values were . (TIF) [file pcbi.1002430.s005.tif]

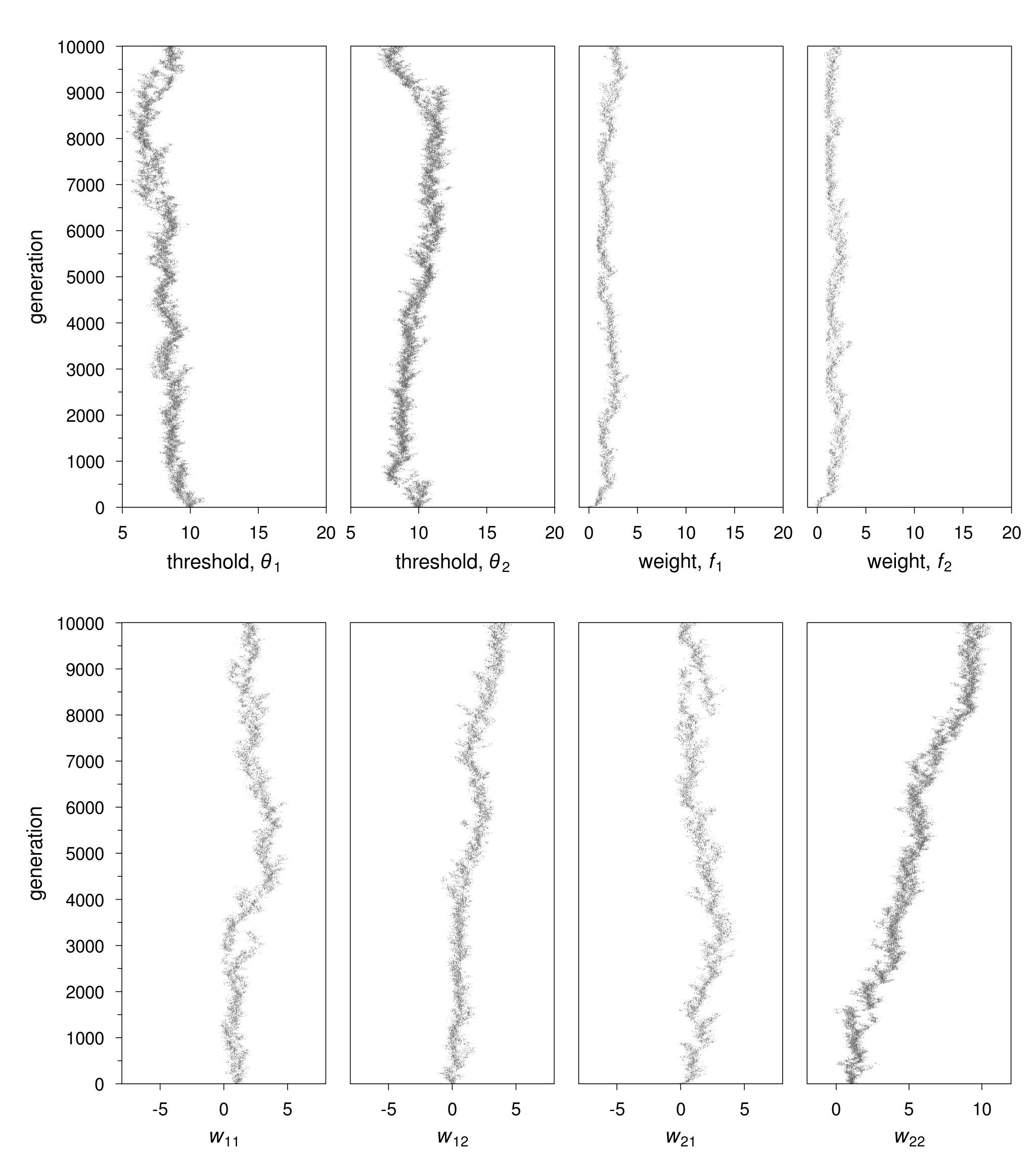

Supplement: Figure S7 — Evolutionary trajectories of thresholds and connection weights of recurrent networks, in a simulation with , and , corresponding to Figure 5A in main text. (TIF) [file pcbi.1002430.s007.tiff]

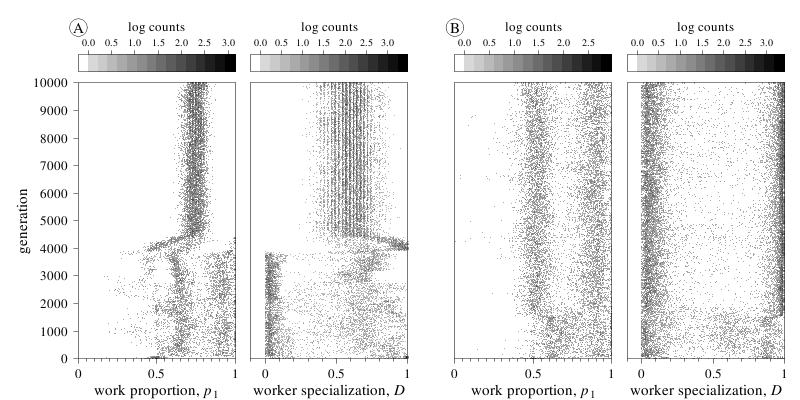

Supplement: Figure S8 — Evolutionary dynamics of two simulations of the evolution of a recurrent neural network, with self-feedback, for , and . The simulations are examples of the two strategies that evolved in response to switching costs. (A) The less frequent outcome (2 out of 10 simulations), where all colonies show values of p 1 close to 0.75, the optimal value, and most colonies show , at the end of the considered evolutionary time. (B) The more frequent outcome, where approximately half the colonies showed around 0.5 and , and the other half showed and . (TIFF) [file pcbi.1002430.s008.tiff]

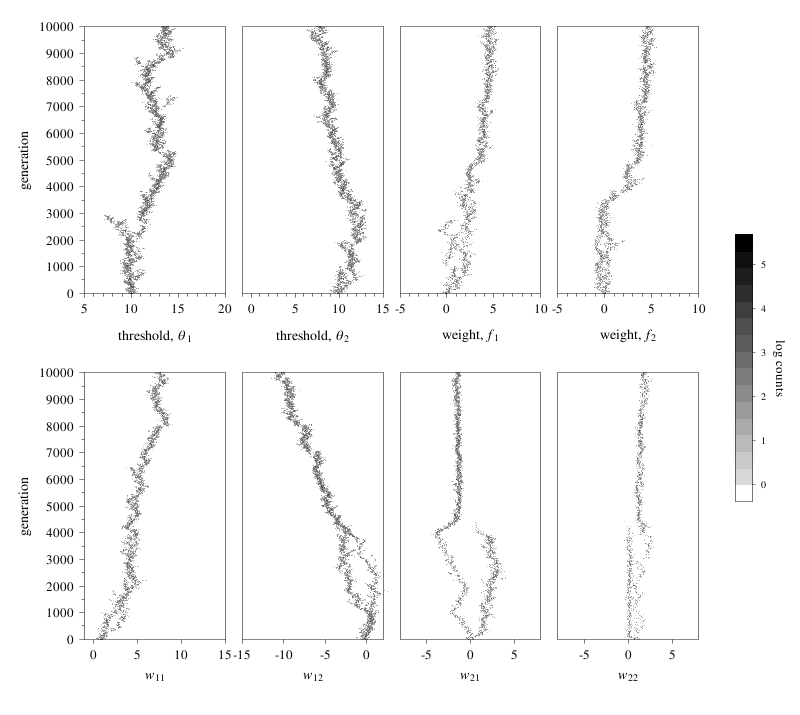

Supplement: Figure S9 — Evolutionary trajectories of thresholds and connection weights of recurrent networks, in a simulation with , and , corresponding to fig. S7A. Top graphs: self-feedback connection weights evolve positive values, as in other simulations where all colonies showed high degree of worker specialization. Evolution of thresholds did not show a specific pattern across simulations, hence it plays a less important role in the outcome. Weights showed positive values for direct connections (with ) and ) and negative values for cross-connections (with ), a pattern also representative for other simulations where all colonies evolved worker specialization. (TIFF) [file pcbi.1002430.s009.tiff]

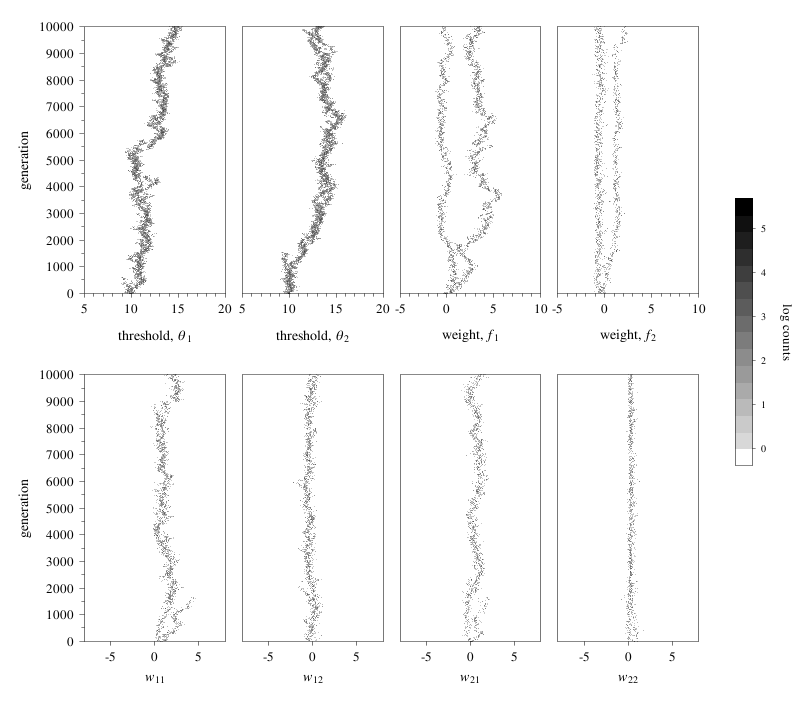

Supplement: Figure S10 — Evolutionary trajectories of thresholds and connection weights of recurrent networks, in a simulation with , and , corresponding to fig. S7B. Top graphs: Self-feedback connection weights go through evolutionary branching, as in other simulations where only a portion of the colonies shows high degree of worker specialization. One branch has positive values, and the other negative values. Bottom graphs: weights are maintained at quite low values, oscillating around zero. (TIFF) [file pcbi.1002430.s010.tiff]
