# Supplementary material for: Implications of Behavioral Architecture for the Evolution of Self-Organized Division of Labor
Source: PLoS Comput Biol. 2012 Mar 22;8(3):e1002430. doi: 10.1371/journal.pcbi.1002430 (PMC3310710; doi:10.1371/journal.pcbi.1002430)
Supplement: Text S1 — Feedforward neural network. (DOC) [file pcbi.1002430.s011.doc]

**Supplementary Information**

**Text S1 - Feedforward neural network**

**Worker specialization and work distribution when**

As shown in the main text (fig. 2), the feedforward neural networks have several limitations. One of them is that work proportion tends to be biased towards one of the tasks, instead of matching the optimal value ($p_{1}=\beta$).

In fig. S1 we show the evolutionary trajectories of the thresholds in the neural network in a representative simulation (also represented in fig. 2A). In agreement with what can be observed in fig. 2A of the main text, where the proportion of work is biased towards task 2, the threshold for task 2, $\theta_{2}$ decreases over evolutionary time in the absence of switching costs (fig S1A).The threshold for task 1, $\theta_{1}$, seems to be changing only due to drift. This may further facilitate the performance of task 2, but the main determinants of the network’s behavior were the connection weights, particularly the cross-connection weights. In fig. S2, we show the stimulus and worker dynamics in a typical colony during the work phase, in the last generation of an evolutionary simulation (corresponding to fig. 2A and S1A). The number of workers engaged in task 2 are, on average, 66.7±4.1 (mean ± sd over the last 90 time steps). On average 33.3±4 workers perform task 1. Stimulus levels for both tasks reflect this: while stimulus 1 reaches higher levels (due to fewer workers removing this stimulus), stimulus 2 is maintained at 1. This value is exactly the value of stimulus increase which is added every time step (we account stimulus values after all changes in stimulus take place). Hence, the stimulus which is added every time step is completely removed. From previous work in the response threshold model, from where we adopted our assumptions on within-colony dynamics, we know that the added stimulus, $\delta$, is removed when $\delta/\alpha$workers are engaged in the task (see Duarte *et al*., subm.). Since worker task choice happens sequentially and stimulus is immediately affected by the work performed, many workers in our simulation inevitably choose task 2 when $S_{2}=0$. This is made possible by the fact that a positive weight connected stimulus 1 to output node 2 (as in fig 2A), hence motivating individuals to keep working for task 2 when stimulus 1 is high.

The limitations of the feedforward neural network prevent the colonies from achieving the maximum possible fitness. Fig. S3 illustrates the magnitude of this deviation. Maximum fitness is achieved when workers are never idle and distribute over tasks according to $A_{ 1}=\beta N$ and $A_{ 2}=(1-\beta)N$. If $A_{ 1}$ and $A_{ 2}$ are the actually observed averages of the numbers of workers performing each of the two tasks per time step, relative colony fitness (*i.e.*, colony fitness divided by maximum fitness is given by:

$\frac{W}{ W_{\max}}=\left(\frac{A_{ 1}}{\beta N}\right)^{\beta}\cdot\left(\frac{A_{ 2}}{(1-\beta)N}\right)^{1-\beta}$ (S1)

This value is plotted in fig. S3 as a function of worker specialization,$D$. In order to minimize initialization effects, the averages $A_{ 1}$ and $A_{ 2}$ were calculated on basis of the last 90% of the work phase. Figure S3 shows that in the absence of switching costs (top left-hand graph) colonies are penalized for not reaching the optimal work distribution $p_{1}=0.5$ (see fig. 2 in the main text and fig. S2).

In the presence of switching costs, colonies are also unable to reach the optimal work proportion of 0.5 (fig. 2B in main text). Hence, colonies are penalized in fitness (fig. S3, top right-hand graph) for not having the optimal work proportion and for the enforced periods of idleness associated with switching between tasks. There is nevertheless a clear, yet weak, relationship between worker specialization and fitness, indicating that worker specialization allows colonies to recover some of the fitness lost due to switching costs.

**Worker specialization and work distribution when** $\beta\text{ = 0.75}$

When the optimal work proportion is biased towards one task and $c=0$, colonies are able to reach maximal fitness (fig. S3, bottom left graph), because they can evolve the optimal work proportion and eliminate idleness. When switching costs are present, worker specialization could not evolve (with recombination). However, individuals could still avoid some of the switching costs by performing mostly task 1. Fitness levels are thus not as low as one could expect (fig. S3, bottom right graph).

**Role of recombination**

Changing recombination rate had an important effect in the outcome of the model. In this sub-section consider only . In the absence of recombination, worker specialization evolved more easily, from $c\geq1$. Higher levels of worker specialization were also obtained than in the presence of recombination (fig. S4B, top panel). All the connection weights of the neural networks showed evolutionary branching (fig. S4B, bottom panel), whereas in the presence of recombination this only occurred for one of the connection weights.

Since all connection weights showed evolutionary branching, evolved networks can differ more strikingly than in the presence of recombination. In fig. S5, we show the parent networks of a highly specialized colony, at the last generation of an evolutionary simulation, under switching costs. The behavior of the networks is remarkably complementary, with the male network specializing in task 2 and the female network in task 1.
