# Supplementary material for: Implications of Behavioral Architecture for the Evolution of Self-Organized Division of Labor
Source: PLoS Comput Biol. 2012 Mar 22;8(3):e1002430. doi: 10.1371/journal.pcbi.1002430 (PMC3310710; doi:10.1371/journal.pcbi.1002430)
Supplement: Text S2 — Recurrent neural networks, with self-feedback. (DOC) [file pcbi.1002430.s012.doc]

**Supplementary Information**

**Text S2 - Recurrent neural networks, with self-feedback**

**Worker specialization and work distribution when** $\beta\text{ = 0.5}$

When the possibility for a self-feedback is included in the network architecture, many of the limitations of the previous architecture are eliminated. In the absence of switching costs, colonies always evolve positive connection weights and self-feedback (fig. S7). In the presence of recombination, it is now possible for all colonies to reach the highest possible fitness, both when switching costs are absent and present (fig. S6, top graphs). The variation found in fitness under switching costs (fig. S6, top right) can be explained by a larger number of colonies deviating from the optimal work proportion (fig. 5B in main text).

In the absence of recombination, however, the most common evolutionary outcome is similar to that observed with the feedforward network. Only a part of the population shows specialization (fig. S6, bottom right). A similar result to that obtained in the presence of recombination, where all colonies show high worker specialization, can only be found at higher switching costs ($c\geq3$), in a minority of the replicate simulations. In fig. S6 (bottom graphs) we show fitness values, plotted against $D$, in two simulations, one where all colonies obtained high $D$, the other where only a part of the colonies obtained high $D$. It can be seen that mean population fitness is lower in the simulations where $D$ is low for a part of the colonies.

**Worker specialization and work distribution when** $\beta\text{ = 0.75}$

When $\beta\text{ = 0.75}$ and $c=0$, colonies can also evolve easily the optimal work proportion, as shown in the main text. In the presence of switching costs, the outcome depended on whether there was recombination or not. In the presence of recombination, evolution of worker specialization rarely occurred. In the absence of recombination, worker specialization was again achieved via two different strategies. In a few simulations, all colonies showed $D\geq0.5$, and obtained values of $p_{1}$ near the optimal, as in the example of fig. S8A. In the majority of simulations, populations showed two types of behaviour (fig. S8B): work more often for task 1, showing no worker specialization; or work for the two tasks in the same proportion, and divide labour, with high worker specialization. The first strategy, where all colonies show relatively high $D$, is obtained by increasing the self-feedback connection weights (fig. S9), whereas the second strategy results from evolutionary branching of self-feedback connection weights (fig. S10). Contrasting with what occurs for $\beta=0.5$in the absence of recombination, the other connection weights do not typically branch in these simulations.
